# Targeted versus tailored multimedia patient engagement to enhance depression recognition and treatment in primary care: randomized controlled trial protocol for the AMEP2 study

**DOI:** 10.1186/1472-6963-13-141

**Published:** 2013-04-17

**Authors:** Daniel J Tancredi, Christina K Slee, Anthony Jerant, Peter Franks, Jasmine Nettiksimmons, Camille Cipri, Dustin Gottfeld, Julia Huerta, Mitchell D Feldman, Maja Jackson-Triche, Steven Kelly-Reif, Andrew Hudnut, Sarah Olson, Janie Shelton, Richard L Kravitz

**Affiliations:** 1 UC Davis Department of Pediatrics and Center for Healthcare Policy and Research, 2103 Stockton Blvd Suite 2224, Sacramento, CA, 95817, USA; 2 UCDHS Clinical Affairs, 2315 Stockton Blvd., Sherman Bldg, Suite 1300, Sacramento, CA, 95817, USA; 3 UC Davis Department of Family and Community Medicine, 4860 Y Street, Suite 2300, Sacramento, CA, 95817, USA; 4 UC Davis Center for Healthcare Policy and Research, 2103 Stockton Blvd Suite 2224, Sacramento, CA, 95817, USA; 5 University of California, San Francisco, 1545 Divisadero, Suite 315, San Francisco, CA, 94143-0320, USA; 6 VA Northern California Health Care System, UC Davis School of Medicine, 10535 Hospital Way-116, California, Mather, 95655, USA; 7 Kaiser-Permanente Northern California, The Permanente Medical Group-Sacramento/Roseville, 2025 Morse Avenue, Sacramento, CA, 95825, USA; 8 Sutter Medical Research Institute, 2801 Capitol Avenue, Suite 400, Sacramento, CA, 95816, USA; 9 University of California, San Francisco, 530 Parnassus Avenue, Suite 366, San Francisco, CA, 94143, USA; 10 UC Davis Division of General Medicine, 4150 V. Street, Suite 2400 PSSB, Sacramento, CA, 95817, USA

**Keywords:** Depression, Primary care, Health communication, Tailoring, Targeting, Patient engagement

## Abstract

**Background:**

Depression in primary care is common, yet this costly and disabling condition remains underdiagnosed and undertreated. Persisting gaps in the primary care of depression are due in part to patients’ reluctance to bring depressive symptoms to the attention of their primary care clinician and, when depression is diagnosed, to accept initial treatment for the condition. Both targeted and tailored communication strategies offer promise for fomenting discussion and reducing barriers to appropriate initial treatment of depression.

**Methods/design:**

The Activating Messages to Enhance Primary Care Practice (AMEP2) Study is a stratified randomized controlled trial comparing two computerized multimedia patient interventions --- one targeted (to patient gender and income level) and one tailored (to level of depressive symptoms, visit agenda, treatment preferences, depression causal attributions, communication self-efficacy and stigma)--- and an attention control. AMEP2 consists of two linked sub-studies, one focusing on patients with significant depressive symptoms (Patient Health Questionnaire-9 [PHQ-9] scores ≥ 5), the other on patients with few or no depressive symptoms (PHQ-9 < 5). The first sub-study examined effectiveness of the interventions; key outcomes included delivery of components of initial depression care (antidepressant prescription or mental health referral). The second sub-study tracked potential hazards (clinical distraction and overtreatment). A telephone interview screening procedure assessed patients for eligibility and oversampled patients with significant depressive symptoms. Sampled, consenting patients used computers to answer survey questions, be randomized, and view assigned interventions just before scheduled primary care office visits. Patient surveys were also collected immediately post-visit and 12 weeks later. Physicians completed brief reporting forms after each patient’s index visit. Additional data were obtained from medical record abstraction and visit audio recordings. Of 6,191 patients assessed, 867 were randomized and included in analysis, with 559 in the first sub-study and 308 in the second.

**Discussion:**

Based on formative research, we developed two novel multimedia programs for encouraging patients to discuss depressive symptoms with their primary care clinicians. Our computer-based enrollment and randomization procedures ensured that randomization was fully concealed and data missingness minimized. Analyses will focus on the interventions’ potential benefits among depressed persons, and the potential hazards among the non-depressed.

**Trial registration:**

ClinicialTrials.gov Identifier: http://NCT01144104

## Background

Depressed patients experience impairment in multiple domains of functioning and well-being [1-7], poorer medical outcomes from chronic conditions [8,9], and increased health care costs [10-12]. Despite improvements in physician training and in systems integrating mental health and primary care, depression remains underdiagnosed in the primary care setting [13-16], the *de facto* mental health treatment system for adults [17-20]. Mild depression is more likely to be overlooked, but a study of undetected cases found that 53% met criteria for major depression one year later [21]. Even when diagnosed promptly, depression is often under-treated [22-25]. These gaps in care may partly be due to patients being reluctant to bring depressive symptoms promptly to the attention of primary care clinicians and to accept initial treatment for depression when it is offered.

Stigma associated with depression is a major barrier to effective diagnosis and treatment in primary care. Silence in the face of depression can be an effective way of avoiding stigmatization and its consequences, including social rejection, overt discrimination, and personal shame [26-29]. Some groups, such as African-Americans, Latinos, and men, are less likely than others to seek care for depression, due in part to such factors as greater perceived stigma and poorer access to high quality health care [25,30-40].

To address these primary care barriers to patient discussion of and initial acceptance of treatment for depression, we developed two alternative communication interventions designed for use by primary care practices [41-44]. The first intervention was a brief, targeted public service announcement (PSA) modeled after direct-to-consumer advertisements for antidepressant drugs but free of commercial bias. The second was a longer, theory-driven, tailored interactive multimedia computer program (IMCP). The overarching objective of this study was to compare the effects of these interventions and an attention control intervention on depression-related behaviors, care processes, and outcomes.

## Methods/design

### Overview of RCT design

The Activating Messages for Enhancing Primary Care Practice (AMEP2) Study was designed as a multicenter, stratified, parallel-group randomized controlled trial comparing three arms: a targeted patient intervention, a tailored patient intervention, and an attention control. Consenting patients were randomly assigned to and viewed an intervention just before a scheduled primary care office visit during which they also completed a previsit survey that included items used for tailoring/targeting, Patients also completed surveys immediately after this *index visit* and at 12-weeks follow-up. Physicians completed brief reporting forms after each patient visit. Additional data were obtained from medical record abstraction and (in a subsample) visit audio recordings.

We conceptualized the trial as two linked sub-studies, one focusing on patients with significant depressive symptoms (Patient Health Questionnaire-9 [PHQ-9] scores ≥ 5) [45], the other on patients with few or no depressive symptoms (PHQ-9 scores < 5). As described below, Sub-study 1 focuses on intervention effectiveness, Sub-study 2 on potential hazards (clinical distraction and overtreatment).

### Description of interventions

In the developmental phase of the project [41-44], we produced two new experimental interventions for improving the detection and treatment of depression in the primary care setting. Each intervention was grounded in a particular approach to creating educational experiences that are relevant to groups and individuals.

The public service announcement (PSA) intervention used *targeted* health messages. Targeted health messages involve segmenting a general population into smaller, more homogeneous units [46]. Typical segments are based on one or more readily observable factors such as age, sex, race or ethnicity, income level, occupation, area of residence, or medical history. Effective targeting, commonly used in social marketing campaigns, uses formative research, such as focus groups, to segment audiences and devise targeted persuasion strategies that are pre-tested for impact. Effectiveness is greatest when the messages emphasize the positive, short-term benefits of change and personal relevance to the listener [47]. Effectiveness is enhanced further when the campaign uses positive role models (such as individuals treated successfully for depression) to enhance social learning and reinforces messages with direct interpersonal influence from credible peers or leaders. The PSAs used in this RCT were targeted to patient gender and income level and were designed to encourage patients to seek depression care and request information (such as whether depression could constitute a reasonable explanation for the presenting symptoms) or diagnostic or therapeutic action (such as prescribing an antidepressant or referring to a mental health professional [48]).

The interactive multimedia computer program (IMCP) used *tailored* health messages. Such methods use information elicited from the respondent, often through a computerized interface, in crafting messages specific to that respondent [49]. In this way, the process of tailoring essentially segments a target audience even further, to the individual level. A growing body of evidence suggests that interventions that are personally tailored to individual mediators or modifiers of health behavior are superior to non-tailored interventions in improving various health behaviors and outcomes across a broad array of patient populations and target conditions [50-55], including depression in primary care [56]. Studies have shown that tailored health messages are better remembered, read, and perceived as relevant [57]. The IMCP provided descriptive and evaluative feedback tailored to the patient based on the following constructs from the previsit survey: level of depressive symptoms [45]; visit agenda (intention to request treatment vs. intention only to discuss depression vs. no intention to discuss depression); treatment preferences; depression causal attributions; communication self-efficacy and depression-related stigma.

Tailored and targeted messages increase the chances of engaging audiences with relevant messages, of creating stronger intentions in recipients to engage in behavior change, and of producing faster and longer-lasting behavioral results. In particular, they can enhance self-efficacy, or the perceived ability to complete a specific task, such as seeking medical care for depression. Perceived barriers to change and the readiness to change can be addressed by reminding recipients of their success in changing their own behavior and by sharing with them the experiences of their normative group.

Individuals randomized to the control intervention viewed a 2.9-minute publicly available educational video on insomnia with a focus on sleep hygiene. This video was chosen as the control because it was comparable in length and format to the experimental PSA and focused on sleep problems that are commonly seen among both depressed and non-depressed patients in primary care.

### Outcome measures for depressed and Non-depressed Sub-studies

AMEP2 consists of two linked sub-studies with the samples drawn from a study population partitioned by the level of depressive symptoms.

### Sub-study 1: patients *with* clinically significant depressive symptoms

This sub-study involves a sample of approximately 500 patients with a Patient Health Questionnaire-9 (PHQ-9) score of ≥ 5 (mild or greater depressive symptoms) on the previsit survey.

**Key Outcome: Initial Depression Care** The key process of care measure is a composite outcome termed *Components of Initial Depression Care (CIDC).* CIDC is coded positive if an antidepressant prescription or mental health referral is provided at the index study visit among the subset of participants with a high likelihood of clinically significant depression (defined as a baseline PHQ-9 score ≥ 10). This outcome is defined as “key” based on the primary study goal, which is to increase patient engagement in care at the initial visit. For patients with clinically significant depression, evidence supports the use of antidepressants and/or psychotherapy or counseling [58-60], and the study was powered to detect a significant intervention effect on this outcome. It is hypothesized that the active interventions will result in a greater likelihood that physicians will offer and patients will accept these treatments.

**Other outcomes** Other outcomes pertinent to Sub-study 1 include effects of the two active interventions on primary care follow-up interval; self-reported physical and mental health at 12 weeks; patient engagement with care during and immediately after the index visit and patient self-efficacy for communication immediately after the index visit; depression-related stigma immediately after the index visit and at 12 weeks; and suicide inquiry by the physician during the index visit.

(1) *Effect of the intervention on primary care follow-up interval.* Based on consensus of the study investigators and advisors, we define a minimally acceptable return visit interval for those not offered antidepressants or a mental health referral as within 2 weeks for those with a PHQ-9 score >14 (consistent with at least moderate depression) and within 4 weeks for those with a PHQ-9 score in the interval from 5 to 14. The former group is dominated by patients with clinically significant depression of more than moderate severity but may include some “false positives”; the latter group consists of a diverse mix of patients including many with dysthymia and sub-syndromal depression [61]. Therefore, in many circumstances, timely followup and careful re-evaluation, rather than immediate antidepressant therapy or referral, may represent good care. Because of the scant evidence base and difficulty in determining the reason for the follow-up interval, this outcome was not included as part of CIDC (the key outcome identified above). It is hypothesized that the active interventions will result in greater adherence to the acceptable follow-up intervals.

(2) *Effects of the interventions on depression severity and self-reported health at 12 weeks follow-up*. These outcomes (using the PHQ-8 [61,62] for depressive symptoms and the SF-12 [63] for self-reported physical and mental health) are based on telephone surveys of patients. It is hypothesized that the active interventions will result in greater improvement in patient functional status at 12 weeks.

(3) *Effects of the interventions on patient self-efficacy for communication with the physician and on engagement with care.* Self-efficacy for communication with the physician is assessed using a 6-item scale modified from the perceived efficacy in patient-physician interactions (PEPPI) scale of Maly et al [64]. The modifications were to modify the wording of the 5-item version of the PEPPI scale to be specific to “mental health concerns” and to add an additional item, worded “*How confident are you that you could talk about depression with a doctor if you wanted to?”* Engagement with care is assessed based on patient-reported readiness for discussion of depression, actual discussions of depression, and requests for information about and treatment for depression. It is hypothesized that the active interventions will result in greater patient communication self-efficacy and engagement.

(4) *Effects of the interventions on depression-related stigma*. We assess this outcome using a 5-item scale modified from Kantor’s Depression Self-Stigma Scale [65]. The underlying hypothesis is that depression-related, patient-centered education and support as delivered by the PSA and IMCP will reduce depression-related stigma.

(5) *Suicide inquiry.* Our prior research suggests that for patients with significant evidence of depression, there is a reluctance by physicians and their patients to discuss self-harm and suicide ideation [66]. By reducing the patient barrier to discussing depression, it is hypothesized that the active interventions will result in increased suicide inquiry by the physician.

### Sub-study 2: patients *without* clinically significant depressive symptoms

This sub-study involves a sample of approximately 300 patients whose baseline PHQ-9 scores were less than 5. The aims of this sub-study are:

(1) To examine whether the interventions are associated with (a) detrimental *process of care* effects (including unnecessary initial antidepressant prescriptions, physician rating of greater effort during visit, longer visit duration), and/or (b) decrements in patient functioning (specifically, worse physical health at 12 week follow-up). It is hypothesized that the active interventions will not result in detrimental outcomes in those without evidence of significant depression.

(2) To examine whether the interventions may lessen perceived depression stigma among non-depressed persons. It is hypothesized that the active interventions will result in reduced stigma after the index visit among patients without evidence of significant depression.

### Measures

Data are obtained from patient questionnaires, physician questionnaire after completed encounter, and encounter chart review. For several measures, similar information is gathered from all three sources. Literature on agreement among these three sources of information in a variety of settings typically suggests only modest agreement [67-70]. For most assessments, the primary measurement source will be considered patient report, which may be more closely associated with outcomes [68]. Based on the theoretical framework of the intervention, the effects of the intervention will depend on the extent to which patients perceive (and recall) specific aspects of encounters. Thus, even if the physician correctly recalls an aspect of the encounter (for example a recommendation for treatment), it is still critical for that recommendation to be perceived by the patient as sufficiently salient to result in patient recall of the recommendation. Chart documentation presumably reflects issues thought important enough by the doctor to document in the chart. But again, unless those issues are effectively communicated to the patient they are less likely to affect patient recall.

The outcome measures, measurement points, and assessment methods for the two sub-studies are summarized in Table 1. When a construct is also measured at baseline (pre-intervention), the same variable definitions are used.

**Table 1:** RCT Sub-studies and Process and Outcome Measures

	**Measurement points**		**Method(s) of measurement**
	**Immediately after index office visit**	**12 week follow-up**	
**Sub-study 1: patients*with*clinically significant depressive symptoms (baseline PHQ-9 ≥ 5)**			
Process of care			
Key outcome - components of initial depression care (CIDC): antidepressant prescription and/or mental health referral among participants with baseline PHQ-9 ≥ 10*	X		PQ, PCPQ, MRR
Others			
Antidepressant prescription and/or mental health referral among all sub-study participants*	X		
Follow-up with PCP at an appropriate interval†	X		
Inquiry regarding self-harm/suicidal thoughts	X		
Patient function			
PHQ-8^‡^ score	X	X	PQ
SF-12 Mental Component Summary score		X	
SF-12 Physical Component Summary score		X	
Patient engagement in depression care			
Readiness			PQ
To discuss depression (in general) with PCP	X		
To discuss depression treatment with PCP	X		
Self-efficacy			
For recognizing depression	X	X	
For discussing depression with PCP	X	X	
Provider-patient interaction			PQ, PCPQ, MRR
Discussion of depression	X		
Patient request for depression treatment	X		
Patient perceived depression stigma	X	X	PQ
**Sub-study 2: patients*without*clinically significant depressive symptoms (baseline PHQ-9 < 5)**			
Process of care			
Antidepressant prescription (over-treatment)	X		PQ, PCPQ, MRR
Distraction effects			
Index office visit length	X		
Patient request for depression treatment	X		
Physician visit effort	X		
Patient function			
SF-12 Physical Component Summary score (potential toxicity, i.e. reduced function)		X	PQ
Patient perceived depression stigma (potential reduction)	X	X	PQ

* Sub-study 1 primarily powered to examine this outcome; research evidence indicates PHQ-9 scores ≥ 10 are associated with significant life impairment (e.g. reduced functional abilities) and a strong likelihood of clinical depression, while scores of 5–9 are more equivocal in this regard.

^†^ Not an evidence-based element of care, but endorsed in prevailing clinical practice guidelines.

^‡^ Includes all PHQ-9 items except the item concerning self-harm thoughts.

Abbreviations:

*AMEP2*, Activating Messages for Enhancing Primary Care Practice; *MRR*, medical record review; *NA*, not applicable (participants in this sub-study are not followed up); *PCP*, primary care provider; *PCPQ*, PCP questionnaire; *PHQ*, Patient Health Questionnaire; *PQ*, patient questionnaire.

### Participants: setting, eligibility, recruitment and institutional review board requirements

**Figure 1:**
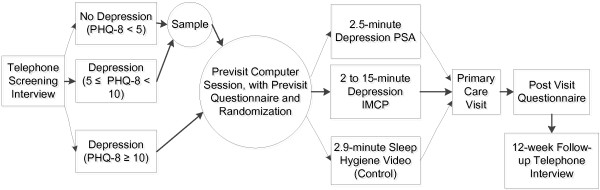
Study design.

We chose to test the targeted and tailored interventions in clinical settings because they are logistically convenient and permit greater investigator control over the intervention. Additionally, patients who are already seeking care for other reasons are closer to the “decision point” for depression care-seeking, where social marketing interventions are likely to be more effective. Figure 1 illustrates the path patients followed from eligibility screening to 12-week follow up, which is described below.

Patients and physicians were recruited from primary care clinics affiliated with two large academic medical centers: University of California (UC) Davis and UC San Francisco (UCSF). While the UCSF performance site primarily involved academic primary care clinics, the UC Davis site offered a mix of academic clinics (where full-time faculty and residents share a practice) and urban and suburban primary care network clinics (where most practitioners are full-time primary care clinicians). Additional performance sites were added to contribute to the diversity of the patient sample; these included adult primary care clinics affiliated with Kaiser Permanente, the United States Department of Veterans Affairs (VA) Northern California Health System, and the Sutter Medical Group (all in Sacramento), plus the San Francisco VA Medical Center.

The AMEP2 study obtained local ethics committee review and approval by the institutional review board (IRB) of record for each study site, including the UC Davis Institutional Review Board (Protocol # 214193); the UCSF Human Research Protection Program Committee on Human Research (Reference #041503, which also covers the San Francisco VA); the IRB/Human Studies Subcommittee of the Department of VA, Northern California Health System (Protocol # 10-10-00600); the Kaiser Permanente Northern California IRB and the Sutter Health Central Area IRB (Reference #226984). The Kaiser Permanente and UC Davis institutional review boards permitted research assistants (RAs) to call patients directly from the appointment lists of consented doctors. At the Sutter Medical Group, the VA sites, and UCSF, the IRBs required that RAs first introduce the study with a letter from the performance site’s principal investigator to prospective research subjects who were scheduled for an upcoming primary care visit. Patients receiving the letter were invited to opt out of the study by telephone within 7 days of receiving the letter. Patients who chose not to opt out were called by RAs to assess interest and eligibility to participate in the study.

Primary care (internal medicine and family practice) physicians and residents in their second and third years of training were eligible for the study and were recruited through email announcements and in-person presentations. Physicians were asked to allow the research team to enroll up to 12 of their patients; they were also asked to complete a brief post-visit questionnaire about the content of the index visit for each patient participating in the study and ideally agree to have the visit audio recorded. Physicians who signed informed consent generally provided blanket consent for their patients to be contacted regarding the study. One physician participant asked to screen all her prospective research subjects before research assistants (RAs) initiated contact.

### Procedures for telephone screening interview for eligibility and sampling of patients

Primary screening for patient eligibility was conducted using a short telephone interview administered one to two weeks before the patient’s next scheduled primary care visit. Patient eligibility was based on: the ability to read and understand English; the ability to use a computer program, and the patient’s self-report that he/she was not currently taking medication for depression. In order to enrich the sample with more severely depressed patients, all patients with a PHQ-8 score of 10 or more were invited to participate. For those patients having a PHQ-8 score of less than 10, a custom programmed computer software application was used to randomly sample patients for inclusion in the study. Patients were sampled independently with varying probabilities (Poisson sampling) according to their PHQ-8 score category and the calendar time of screening, with the sampling probabilities updated occasionally to help ensure that the study would accrue the target total sample of 308 in the non-depressed group. Eligible patients who verbally agreed to participate in the study at the end of the screening interview were invited to a research appointment on the day of their scheduled primary care visit.

### Procedures on Day of office visit: prior to randomization

Patients were asked to meet the RA in their primary care physician’s waiting room one hour before their scheduled visit. Upon meeting the patient, the RA accompanied the patient to a private room or a quiet alcove of the waiting room to conduct informed consent and complete the intervention.

Informed consent was obtained in-person by the RA during the research appointment. RAs explained all research procedures, including the use of computerized surveys before and immediately after the doctor’s visit, the importance of audio recording the visit, the possibility of being contacted for a 12-week follow-up interview, and procedures for obtaining study payment (a $20 gift card at the index visit and a $10 gift card for the 12 week follow-up) . Patients had to provide informed consent and sign a Health Insurance Portability and Accountability Act (HIPAA) release, thereby allowing the research team access to the primary care physician’s progress note for the visit, in order to continue with the study. Patients were also asked to provide their consent to have their doctor visit audio recorded when the physician provided prior consent for this component of the study, which was presented as being “optional” to both physicians and patients. At least one audio recording was collected from 81 of the 135 physicians (60%) participating in our study. Patients who successfully completed the informed consent process were logged on to a tablet PC for randomization and intervention assignment. When the intervention was conducted in a waiting room, patients were provided with a headset to keep the multi-media computer intervention private and to minimize distractions associated with busy waiting rooms.

### Randomization

The unit of randomization was the patient. We chose to randomize individual patients rather than physicians because the intervention was intended to operate at the patient level (affecting patient knowledge, attitudes, self-efficacy, and behaviors) and because the improved precision of estimated intervention effects that arises from randomizing patients from the same physician to different study arms was felt to outweigh the increased risk of bias from between-arm contamination that arises from communication among patients.

During the previsit computer session, patients were randomized to an intervention after answering questions about their gender and race/ethnicity, via a computerized randomization algorithm incorporated into the multi-media computer interface. To ensure balanced assignment by gender and race/ethnicity, the computer randomization program stratified subjects into one of eight groups: black female, black male, Hispanic female, Hispanic male, white female, white male, other female, other male. Computerized racial/ethnic group assignment was based on the following decision rule: 1) Hispanic trumps all other selections, 2) for non-Hispanics, African-American trumps all (e.g., someone selecting African-American and Asian for race would be put into the African-American randomization category); and 3) for people not selecting black, “other” categories trump white (e.g., if someone selects Native American and white, they are randomized as other). Within each of these eight categories, patients were randomly allocated in equal proportions to one of the three study arms, in randomly permuted blocks of nine subjects.

### Procedures on Day of office visit: post-randomization

Immediately after being randomized and prior to revealing the assigned intervention to the patient during the previsit computer session, the patient was queried a second time regarding current use of antidepressant medications, an exclusion criterion for participation in the study that was first assessed and used as an exclusionary criteria during the initial telephone screening. Patients reporting current use to this inquiry were informed that they did not meet eligibility rules for continuing in the study and were thanked for their participation. Assessing this exclusion criterion *after* randomization occurred was a design flaw that resulted in a small fraction of randomized patients being excluded from further study. Notwithstanding this flaw, the exclusions that resulted could not, as a practical matter, have been affected by the randomized assignment and hence we do not expect that this flaw can bias the estimates of between-arm contrasts.

After intervention assignment, patients continued to answer baseline (previsit, preintervention) survey questions for use in targeting/tailoring and to assess baseline health status. Regardless of assignment, all patients answered the same series of questions, including items on income, visit agenda (intention to request treatment vs. intention only to discuss depression vs. no intention to discuss depression); treatment preferences; depression causal attributions; communication self-efficacy and stigma used for tailoring or targeting interventions. The computer software was designed to automatically store responses in a computer database and to use these responses to customize (i.e. target/tailor) assigned interventions accordingly. After completing the pre-visit questionnaire, the patient watched his or her randomly assigned and appropriately customized intervention: a targeted PSA, the tailored IMCP, or the (uncustomized) control video. The PSA and control video were approximately 2.5 minutes in length. Typically, patients assigned to the IMCP spent between 2 (10^th^ percentile) and 15 (90^th^ percentile) minutes viewing educational materials.

After patients viewed the material and exited the computer session, they waited to be called in for their scheduled office visit. Patients who reported recent (<2 weeks) suicidal thoughts were flagged by the computer so that the RA could involve the patient in the decision to notify the physician with a written message about their risk for suicide. The RA did this by delivering a letter to the physician prior to the visit alerting the physician to the patient’s status. Beyond these efforts, study personnel did not attempt to control patient-PCP communication content or force discussion of depression treatment.

### Procedures for post-visit assessments

Immediately following the office visit, subjects returned to the tablet PC to complete a post-visit questionnaire, which contained many of the same items as on the pre-visit questionnaire plus questions about the encounter (e.g., whether they asked about depression and/or depression-related care and whether the physician prescribed an antidepressant, made a mental health referral, or arranged for follow-up). Twelve weeks after the index visit, RAs telephoned patients reporting PHQ-8 scores of ≥ 5 *either* during screening *or* on the day of the index visit to assess treatment adherence, severity of depressive symptoms, and health status using a scripted follow-up interview.

### Power calculations and sample size

By setting target sample sizes of 170 subjects per arm, the effectiveness sub-study was designed to provide 80% power to detect between-arm (pairwise) differences in the proportion receiving CIDC of 15 percentage points and standardized differences of 0.3 standard deviations (sd) in the mean levels of continuous outcomes, under two-sided testing with alpha = 5% [71]. For the sub-study focusing on non-depressed patients, the target enrollment of 102 subjects per arm provides 80% power to reject the noninferiority null hypothesis that exposure to an experimental intervention groups (N = 204 in the PSA/IMCP groups) is associated with at least a 3.5 percentage point increase in the extremely low risk anticipated for antidepressant prescribing in the control group (N = 102), under one-sided testing with alpha = 5% and assuming that the true risk is 1% in each of the three groups [72]. For pairwise comparisons of means, a sample size of 102 in each arm of the non-depressed sub-study provides greater than 80% power to detect differences of 0.4 sd [71].

### Data safety and monitoring plan

Three categories of patient safety concerns apply to this study: 1) heightened anxiety and possible prolonged visit lengths among patients who are led to believe they have depression but do not (false positives): 2) missed diagnoses of depression (false negatives); and 3) appropriate diagnosis and treatment of patients with moderate, moderately severe, and severe depression, including those experiencing suicidal ideation. The study addressed the potential for longer visit length by reminding physicians of the content of the intervention and suggesting supportive language and resources that could be used with patients wanting to discuss their symptoms. No specific provisions were made to deal with missed diagnoses of depression among those with low PHQ-9 scores, as rates of missed depression would be no higher than in usual care. Regarding suicidal ideation among enrolled patients, when patients responded positively to the suicidality question in the PHQ-9, the research assistant: 1) offered the patient three telephone numbers in the respondent’s local calling area; 2) spoke with the patient about notifying the doctor; 3) initiated a confidential letter to the doctor about the patient’s status (hand delivered to the physician before the patient’s scheduled appointment in the clinic); and 4) notified the project manager, who notified the principal investigator about the event within 24 hours. The PI (or, alternatively, the study physician on call) in turn paged the patient’s primary care physician to discuss care of the patient within two working days.

Beyond threats to patient safety, procedures were established regarding the documentation of untoward events, including breaches of confidentiality. Any known breach of confidentiality was to be reported to the principal investigator within 24 hours. The principal investigator was required to notify the appropriate IRB(s) of all untoward events within one week. These events were also meant to be discussed during regular meetings of the data safety and monitoring board.

### Patient accrual and study flow

**Figure 2:**
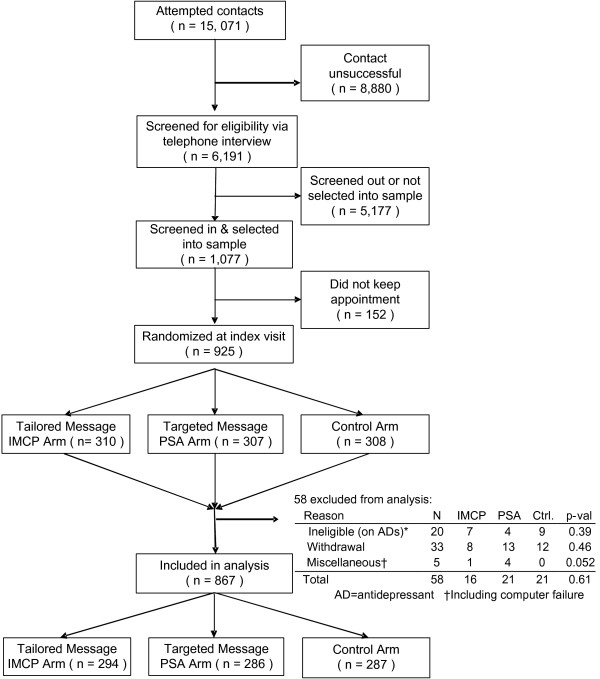
CONSORT diagram.

Altogether, 867 patients were accrued into the study and randomized successfully (Figure 2), 559 with PHQ-9 scores ≥ 5 for Sub-study 1 and 308 with PHQ-9 scores <5 for Sub-study 2 (Table 2). Randomization was generally successful, with modest between-group differences in baseline characteristics (Table 2).

**Table 2:** Characteristics of participating patients

**Characteristic**	**Interactive-multimedia arm (n = 294)**	**PSA arm (n = 286)**	**Attention control arm (n = 287)**	p-value^*^
% female	162 (55.1)	159 (55.6)	165 (57.5)	0.83
Mean age, yrs (SD)	51.5 (12.4)	52.2 (11.4)	51.7 (11.5)	0.82
Race/ethnicity (%)				0.86
Hispanic or Latino	43 (14.6)	40 (14.0)	40 (13.9)	
Black, not Hispanic	61 (20.7)	50 (17.5)	57 (19.9)	
Asian or other	25 (8.5)	32 (11.2)	33 (11.5)	
White	165 (56.1)	164 (57.3)	157 (54.7)	
Income (%)				0.70
Less than $35,000	106 (36.0)	110 (38.5)	101 (35.2)	
$35,000 or more	188 (64.0)	176 (61.5)	186 (64.8)	
% with college or graduate degree	126 (43.3)	123 (43.2)	142 (49.6)	0.20
% living with spouse or partner	162 (55.9)	160 (56.1)	165 (57.7)	0.89
Site of care (%)				0.51
Specialty Practice	119 (40.5)	130 (45.5)	114 (39.7)	
Academic	94 (32.0)	89 (31.1)	105 (36.6)	
HMO	32 (10.9)	30 (10.5)	24 (10.9)	
VA	49 (16.7)	37 (12.9)	44 (15.3)	
PHQ-8 (SD)				
At enrollment	7.9 (5.4)	7.0 (5.1)	7.2 (5.4)	0.11
At index visit	7.9 (5.5)	6.7 (5.3)	7.1 (5.4)	0.05
*Correlation	0.84	0.84	0.86	
PHQ-9 category, N (%)				0.17
<5	90 (30.6)	109 (38.1)	109 (38.0)	
5-9	99 (33.7)	103 (36.0)	89 (31.0)	
10-14	66 (22.4)	43 (15.0)	56 (19.5)	
15+	39 (13.3)	31 (10.8)	33 (11.5)	
SF-12 at enrollment, Mean (SD)				
Mental Health Score (n = 840)	44.8 (11.8)	47.7 (11.6)	46.3 (12.7)	0.02
Physical Health Score (n = 840)	41.4 (13.5)	41.8 (13.7)	41.2 (13.2)	0.86
Somatic Symptom Severity at Index Visit†, Mean (SD)	7.3 (2.0)	7.3 (2.0)	7.4 (1.9)	0.90
Depression Stigma at Index Visit, Mean (SD)	16.5 (4.0)	16.9 (3.8)	16.5 (3.8)	0.42
Self-efficacy for patient-physician interactions (PEPPI) regarding mental health, Mean (SD)	21.9 (5.8)	21.7 (5.6)	21.8 (5.9)	0.86

^*^To obtain p-values, the F-test statistic in a one-way ANOVA was used for comparing means and the chi-square test for association was used for categorical variables.

†Reflects how much patient was bothered in the past month (theoretical range from 4 to 12) by stomach pain; back pain; pain in arms, legs or joints, and headaches.

### Planned analytic approach

For all outcomes except for antidepressant behavior in the non-depressed patients, analyses will be implemented using multiple regression models for clustered data to account for the nesting of patient-level observations within providers and, for longitudinally assessed outcomes, the nesting of repeated measures within patients. Most analyses will take the form of generalized linear mixed models [73], with logistic regression models estimated via generalized estimating equations [74] used as a robust backup in case mixed-model assumptions are violated or computational difficulties (lack of convergence) are encountered.

In generalized linear mixed models, random effects will be specified for the physician (to account for the nesting of patients within physician). For longitudinal outcomes, random effects will also be specified for patient. The key independent variable will be the study group assignment (control vs. PSA vs. IMCP) and additional independent variables will be included to account for the stratified study design. Wald tests, point estimates and 95% Confidence Intervals will be used to assess hypothesized contrasts among the study arms.

Because the incidence of antidepressant behavior in the non-depressed patients is expected to be very low and the primary contrast of interest is for testing the one-sided noninferiority hypothesis that the incidence is not 3.5 percentage points higher in the combined PSA/IMCP groups versus the control, we will use restricted maximum likelihood methods to construct a 2-sided 90% confidence interval for the PSA/IMCP vs. Control risk difference in antidepressant prescribing risk to assess this null hypothesis, as described by Farrington and Manning [72].

## Discussion

Among the many challenges in primary care, the initial diagnosis and management of depression is perhaps the one most fraught by patient perceptions and stigma, potentially inhibiting open and effective communication. To address this problem, the RCT described in this protocol was designed to compare two different approaches to patient engagement (i.e. a targeted PSA and tailored IMCP) and an active control (sleep hygiene video) in terms of their ability to help patients to discuss their depressive symptoms with their primary care physicians and reduce stigma about depression. The interventions were developed over a period of two years based on intensive foundational research that included focus groups, a population based survey, and a conjoint analysis [41-44]. In addition to improving initial care for depression, it was viewed as critical to examine effects in patients who were not depressed, to ensure that neither patients nor physicians were distracted from attending to the patients’ other health problems, and to examine whether the interventions would contribute to reducing stigma in this larger patient population. The study was implemented in six practice sites in two Northern California cities. While limited in duration of follow-up and generalizability, this is one of the largest trials of computer-based patient engagement interventions in depression yet conducted.

We decided to deploy the intervention programs in clinic settings. In contrast, pharmaceutical manufacturers have enjoyed considerable success with direct-to-consumer advertising, which exposes a large population repeatedly to messages urging patients to “ask your doctor” about depression and antidepressant therapy [75,76]. While such a strategy might be optimal for our PSA intervention, it would not have been appropriate for the IMCP intervention, which was focused on facilitating patient communication with the physician immediately following an encounter. To enable comparisons among the interventions we used a clinic-based strategy. While it may have been preferred to re-expose subjects to the interventions over time, limited study resources precluded such an approach. However, if the initial effects of the interventions are promising such an approach may be warranted.

This project’s emphasis on initial care of depression is based on evidence that depression in primary care often goes undiagnosed [13], that clinically mild depression not infrequently evolves to major depression within a year [21], and that even detected depression is often under-treated in primary care [22,23]. Although subsequent care (including attention to patient adherence, careful monitoring of depression treatment response, and avoidance of clinical inertia) is critically important, none of this is possible in the absence of accurate diagnosis and appropriate initial care.

In conducting the trial, we encountered several noteworthy challenges and limitations:

•To accrue the 867 patients ultimately enrolled in the study, we attempted to reach more than 15,000 patients by telephone and screened over 6000. In settings where local ethics boards required an opt-out process prior to any telephone contact (allowing patients to return a postcard indicating their desire not to be screened), screening was even less efficient. Yet we were ultimately successful in enrolling a sample that appears representative of the underlying population of interest, at least in the clinics studied.

•Our protocol required a substantial time investment by patients, as they were required to arrive in clinic one hour prior to their scheduled appointment. In a small proportion of encounters, patients arrived too late to administer the intervention and accompanying surveys, and they had to be rescheduled. Most of the pre-visit time required of patients was for informed consent and survey administration; the PSA and control videotapes were less than three minutes in length, and the median time patients spent with the IMCP was 5 minutes.

•Enrollment was limited to English-speaking patients, and English-speaking Latinos constituted less than 10% of the sample. Generalizability of the findings to this important demographic group will be restricted.

In summary, the results of this RCT will address the question of whether multimedia patient engagement programs for depression improve processes and outcomes of care for depressed persons. In addition, the trial will compare the relative effectiveness of targeted versus tailored interventions. Finally, the study will address whether the interventions are “safe” (i.e. that they do not worsen processes or outcomes of care) and could even have benefits (e.g. fostering less stigmatized views of depression) among non-depressed persons.

## Abbreviations

AMEP2: Activating Messages for Enhancing Primary Care Practice; CIDC: Components of Initial Depression Care; HIPAA: Health Insurance Portability and Accountability Act; IMCP: Interactive multimedia computer program; IRB: Institutional review board; PEPPI: Perceived Efficacy in Patient-Physician Interactions; PSA: Public service announcement; PHQ: Patient Health Questionnaire; RA: Research assistant; RCT: Randomized clinical trial; UC: University of California; UCSF: University of California, San Francisco; VA: United States Department of Veterans Affairs

## Competing interests

No competing interests have been declared.

## Authors’ contributions

RLK secured funding. RLK, AJ, PF and MDF and DJT made substantial contributions to the conception and design of the study. RLK, CKS, AJ, PF, CC, DG, JH, MDF, MJT, SKR, AH and SO made substantial contributions to the acquisition of study data. RLK, DJT, JN, JS, CKS, AJ and PF made substantial contributions to the analysis and interpretation of study data. CKS and DJT developed the initial drafts of the manuscript. Substantial revisions were made by RLK, AJ and PF. All authors read and approved the final manuscript.

## Pre-publication history

The pre-publication history for this paper can be accessed here:

http://www.biomedcentral.com/1472-6963/13/141/prepub
